# Amanita-Induced Hepatitis

**DOI:** 10.7759/cureus.72325

**Published:** 2024-10-24

**Authors:** Elisabete Ribeiro, Sara Silva, Marta Batista, Maria Luis Santos, Ana Gonçalves

**Affiliations:** 1 Internal Medicine, Hospital Senhora Oliveira, Guimarães, PRT

**Keywords:** amatoxin, hepatitis, liver failure, mushrooms, n-acetylcysteine

## Abstract

The consumption of wild mushrooms can be associated with poisoning, particularly with species containing hepatotoxic toxins such as amatoxin, present in more than 35 species. We present a case of Amanita-induced hepatitis in a 73-year-old man after ingestion of wild mushrooms. He presented to the emergency department (ED) with severe diarrhea, intense abdominal pain, and vomiting, with onset approximately six hours after ingestion of wild mushrooms. He developed acute liver injury but never developed hepatic encephalopathy. His clinical course was favorable, with complete recovery of liver function. The diagnosis of Amanita-induced toxic hepatitis is based on clinical observations. Early diagnosis and treatment are fundamental factors in the prognosis of these patients.

## Introduction

The ingestion of wild mushrooms containing hepatotoxic substances can be associated with episodes of liver injury. Mushrooms are ubiquitous in nature and commonly found in more humid and shaded areas [1]. It is estimated that there are over 140,000 species of mushrooms in nature, of which only 10% have been identified. Only a small percentage of these have received approval for consumption. Mushroom poisoning occurs when species with hepatotoxic toxins are ingested [2,3]. These can be classified into protoplasmic (the most dangerous), neurotoxins, disulfiram-like reactions, and gastrointestinal irritants. The ingestion of mushrooms containing amatoxin (present in over 35 species) is associated with episodes of acute hepatitis and is responsible for most fatal mushroom poisonings due to the hepatocellular necrosis they cause [1,4].

*Amanita phalloides* is a mushroom that frequently triggers acute hepatitis, leading to numerous hospital admissions and deaths [1,2]. This mushroom is composed of potent toxins - cyclopeptides - large molecular-weight particles divided into phallotoxins, virotoxins, and amatoxins. Amatoxins are the most toxic, responsible in some cases for liver, kidney, and intestinal damage [1,5]. *A. phalloides* poisoning is more frequent in late summer and autumn [2]. The severity of the intoxication depends on various factors, such as the amount of toxin ingested, the early onset of symptoms, and delayed diagnosis with consequent delayed treatment [2,4]. The clinical evolution is variable, ranging from asymptomatic to liver failure [4]. The diagnosis of *A. phalloides* poisoning is clinical, although some analytical techniques such as RIA (radioimmunoassay) and HPLC (high-performance liquid chromatographic method, ELISA) can detect amatoxins in blood or urine up to 48 hours after ingestion. However, these tests are not widely available, and the urinary concentration of α-amanitin does not correlate with the severity of liver damage [4,6]. The treatment of amatoxin poisoning relies on the management of dysfunctions; there is no specific treatment or direct antidote. However, the use of certain substances such as silibinin, N-acetylcysteine, and benzylpenicillin has shown a beneficial effect in the management of these patients, although there is no complete consensus on their isolated or synergistic effect [2,4,7].

## Case presentation

A 73-year-old male with a medical history of hypertension, dyslipidemia, grade 1 obesity, and atrial fibrillation, usually medicated with lisinopril/amlodipine 20/5 mg daily, atorvastatin 40 mg daily, and apixaban 5 mg twice daily, presented to the emergency department (ED) with complaints of diffuse, intense abdominal pain with about 24 hours onset and associated with diarrhea and vomiting. He reported consumption of wild mushrooms the night before admission to the ED.

There was a marked increase in transaminases on the liver profile, with associated elevations in gamma-glutamyl transferase (GGT), lactate dehydrogenase (LDH), and direct hyperbilirubinemia without coagulopathy. In this context, given the worsening analytical results and severe acute liver injury, the Liver Transplant Center was contacted. The patient was admitted to the Intermediate Care Unit due to acute toxic hepatitis secondary to wild mushroom ingestion. Upon admission, the patient presented with multi-organ dysfunction: renal, metabolic, and hematologic dysfunction, and initiated a treatment protocol involving N-acetylcysteine and silibinin.

The patient showed symptomatic improvement during the first three days of hospitalization under a protocol of silibinin, N-acetylcysteine, and supportive care. The vomiting resolved within the first two days of hospitalization, later resolving the abdominal pain. However, the patient experienced a laboratory deterioration. Specifically, there was a marked elevation of transaminases and GGT, as illustrated in Table 1, with a peak of 15365 U/L for ALT and 8321 U/L for AST. After reaching this peak, the transaminases showed a descending profile, with the decrease in AST values being slower than ALT.

**Table 1:** Evolution of transaminases, GGT, and ALP during hospitalization AST: aspartate aminotransferase (N: 12–40 UI/L); ALT: alanine aminotransferase (N: 7–40 UI/L); GGT: gamma-glutamyl transferase (N: 0–73 UI/L); ALP: alkaline phosphatase (N: 46–116 UI/L); ED: emergency department

	ED	D0	D1	D2	D3	D4	…	D9	Discharge
AST (UI/L)	36	2867	15,365	5594	1445	429		68	35
ALT (UI/L)	33	1812	8321	7757	5694	3076		1086	58
GGT (UI/L)	67	88	98	--	288	367		98	125
ALP (UI/L)	67	--	65	62	79	98		98	68

Concomitantly, there was a worsening of liver function (Table 2), with hyperbilirubinemia and an INR greater than 1.5.

**Table 2:** Evolution of bilirubin, albumin, and INR values during hospitalization TD: total bilirubin (N: 0.3–1.2 mg/dL), DB: direct bilirubin (N: 0–0.3 mg/dL), INR: International Normalized Ratio (N: 0.8–1), ED: emergency department

	ED	D0	D1	D2	D3	D4	…	D9	Discharge
TD (mg/dL)	0.68	1.66	2.02	9.34	12.85	12.52		4.29	--
DB (mg/dL)	0.18	0.90	0.93	6.70	9.80	9.40		3.27	--
Albumin	--	4.7	4.1	2.9	2.7				3.8
INR	--	1.1	1.2	1.3	1.6				1

During hospitalization, the patient completed a six-day course of N-acetylcysteine and silibinin, and clinical improvement was observed in the first three days, with vomiting and diarrhea resolved. The patient never developed encephalopathy. Regarding the multi-organ dysfunction at admission, there was a favorable evolution during hospitalization, with all dysfunctions resolved at the time of discharge. At follow-up, two months later, the patient was asymptomatic with a normal liver profile.

## Discussion

Mushroom consumption is associated with frequent poisoning incidents, particularly when consuming species with hepatotoxic toxins. Mushroom poisoning is seasonal, occurring more frequently in late summer and autumn. Over 35 species of mushrooms from the three genera (Amanita, Galerina, and Lepiota) contain amatoxin, the hepatotoxic substance that is stable even at high temperatures [1]. The diagnosis of intoxication is clinical; severe gastrointestinal symptoms, such as profuse vomiting and diarrhea within hours or a day after ingesting wild mushrooms, are suggestive of poisoning [4]. Additionally, measuring amatoxin levels in serum and urine is possible, although these tests are not available in all centers. However, amatoxin measurement is not used transversally and routinely since the urinary concentration of the toxin does not correlate with the severity of liver injury [7]. Analytically, hematological alterations (leukocytosis, thrombocytopenia) and elevation of liver enzymes are the most frequently found changes. Liver enzymes serve as indicators of prognosis and hepatocellular necrosis, while the elevation of total bilirubin and INR signals liver failure, thereby indicating the need for liver transplantation. Thrombocytopenia may appear in amanita poisoning due to bone marrow toxicity and the presence of disseminated intravascular coagulation. Hematuria, glycosuria, kidney injury, hypoglycemia, hyponatremia, and hypernatremia may also be present [2,8,9].

Amanita poisoning is characterized by different stages, ranging from almost asymptomatic evolution to liver failure. The severity of poisoning depends not only on the amount of toxin ingested but also on the time elapsed between ingestion and symptoms and from diagnosis to the start of treatment. Early onset of gastroenteritis, 6-12 hours after mushroom consumption, correlates with more severe hepatotoxicity [5]. In stage I, 6-24 hours after ingestion, there are mainly gastrointestinal symptoms associated or not with kidney injury; in stage II, there is an apparent improvement, with resolution of gastrointestinal symptoms but elevation of liver enzymes; and stage III, which occurs 48-96 hours after ingestion, is characterized by a peak in transaminase elevation (especially between 60 and 72 hours after ingestion), with consequent massive hepatocyte destruction, coagulopathy, and encephalopathy. If the patient is in the first stage/gastrointestinal phase, the essential thing is to correct and/or prevent dehydration, ionic, metabolic, and acid-base alterations, and therefore, intense fluid therapy is necessary. In fact, a urine output of 100-200 ml/hour in the first four to five days increases renal elimination of amatoxins, and forced natriuresis is not necessary [2,4,10].

The treatment of mushroom poisoning is mainly supportive, with support for dysfunctions with intense fluid therapy, for example, and is based on four pillars. The first objective is to decrease the enterohepatic circulation of amanita, and, therefore, gastric lavage with activated charcoal at a dose of 0.5 mg/kg 4/4 hour is recommended if ingestion occurs less than six hours and can be extended up to 24 hours after ingestion [4,7,11]. The second objective involves inhibiting the absorption of amanita into the hepatocyte. Silibinin is a substance with hepatoprotective effects that directly competes with amatoxin for the transmembrane transporter, thus inhibiting the entry of the toxin. It is recommended at an initial dose of 5 mg/kg in a bolus followed by an infusion of 20-50 mg/kg/day if ingestion occurs less than 48 hours and should be maintained for up to six days or until clinical improvement [7,10]. If silibinin is not available within six hours, penicillin use is recommended, but always in association with N-acetylcysteine because together they interrupt the enterohepatic circulation of the toxin and, thus, promote the decrease of its recirculation. As antioxidant therapy, the use of N-acetylcysteine is recommended at the same dose used in paracetamol poisoning. The recommended dose is 150 mg/kg in one hour of loading, and subsequently 50 mg/kg for four hours and 100 mg/kg for 16 hours, and this should be done for five days or until clinical improvement [12,13]. Also, as antioxidant therapy, vitamin C (3 g IV/day) and cimetidine (300 mg IV 8/8 hours) can also be used simultaneously until clinical improvement [10].

There are several techniques for extracorporeal removal of amatoxins; however, their efficacy has not been proven, namely plasmapheresis or MARS (molecular adsorbent recirculating system). The latter corresponds to a dialysis method with albumin that removes primary and secondary toxins, supports renal function, and can be used even before gastrointestinal symptoms. This treatment potentially reduces the number of toxins in circulation, minimizing hepatocellular injury, and can serve as a bridge to liver transplantation [4,6]. The prognosis should be evaluated in a similar way to the models for other hyperacute syndromes, as occurs in paracetamol poisoning.

Data show an overall mortality that can vary from 2% to 20% in adults worldwide, with an estimated mortality of 6.8% in Portugal due to mushroom poisoning. Worse prognosis factors include female sex, age under 10 years, short interval between ingestion and onset of symptoms, as well as signs of liver and kidney dysfunction [3,4,8]. Patients who survive acute liver injury from amanita, apparently without sequelae, progress to chronic liver disease in 20-79% of cases [10].

## Conclusions

Ingestion of mushrooms can lead to frequent poisoning incidents, especially when consuming species that contain hepatotoxic toxins, such as amanita. Despite the possibility of measuring amatoxins, the diagnosis of toxic hepatitis due to amanitas remains clinical, as the severity of the liver injury does not correlate with the urine concentration of the toxin. Previous cirrhosis does not increase the risk of hepatotoxicity, but it may worsen and have a worse prognosis. Toxin-induced liver damage can present in a variety of ways, such as by mimicking biliary tract obstruction or viral hepatitis, for instance. We should closely monitor and promptly treat patients exhibiting signs or symptoms of hepatic impairment.
